# Allicin Induces Calcium and Mitochondrial Dysregulation Causing Necrotic Death in *Leishmania*

**DOI:** 10.1371/journal.pntd.0004525

**Published:** 2016-03-29

**Authors:** María J. Corral, Elena Benito-Peña, M. Dolores Jiménez-Antón, Laureano Cuevas, María C. Moreno-Bondi, José M. Alunda

**Affiliations:** 1 Department of Animal Health, Group ICPVet, Faculty of Veterinary Medicine, University Complutense Madrid, Spain; 2 Department of Analytical Chemistry, Optical Chemosensors and Applied Photochemistry Group (GSOLFA), University Complutense Madrid, Spain; 3 National Microbiology Centre, Institute of Health Carlos III (ISCIII), Majadahonda, Madrid, Spain; Yale School of Public Health, UNITED STATES

## Abstract

**Background:**

Allicin has shown antileishmanial activity *in vitro* and *in vivo*. However the mechanism of action underlying its antiproliferative effect against *Leishmania* has been virtually unexplored. In this paper, we present the results obtained in *L*.*infantum* and a mechanistic basis is proposed.

**Methodology/Principal Finding:**

Exposure of the parasites to allicin led to high Ca^2+^ levels and mitochondrial reactive oxygen species (ROS), collapse of the mitochondrial membrane potential, reduced production of ATP and elevation of cytosolic ROS. The incubation of the promastigotes with SYTOX Green revealed that decrease of ATP was not associated with plasma membrane permeabilization. Annexin V and propidium iodide (PI) staining indicated that allicin did not induce phospholipids exposure on the plasma membrane. Moreover, DNA agarose gel electrophoresis and TUNEL analysis demonstrated that allicin did not provoke DNA fragmentation. Analysis of the cell cycle with PI staining showed that allicin induced cell cycle arrest in the G_2_/M phase.

**Conclusions/Significance:**

We conclude that allicin induces dysregulation of calcium homeostasis and oxidative stress, uncontrolled by the antioxidant defense of the cell, which leads to mitochondrial dysfunction and a bioenergetic catastrophe leading to cell necrosis and cell cycle arrest in the premitotic phase.

## Author Summary

Leishmaniasis is a vectorial parasitic disease caused by flagellate organisms from the genus *Leishmania*. Infection is present in over 80 countries and visceral forms are the second most fatal human parasitic disease. Control relies on chemotherapy but available drugs have important shortcomings such as toxicity, side effects, unaffordable price of the safest presentations and increasing reports of parasite resistance and clinical failures. Thus, new drugs are needed. Allicin, a molecule obtained from garlic, has shown antiproliferative effect against different cancer cells, bacteria, fungi and Protista including *Leishmania*. Insofar its mechanism of action is poorly known. Our results with *L*.*infantum* point towards allicin inducing high levels of intracellular calcium, redox inbalance, and mitochondrial dysfunction with reduction of ATP. These events lead to cell necrosis without evidence of apoptotic-like markers. The proposed model suggests the potential use of allicin against leishmaniasis, alone or in combination with other drugs with different mechanisms of action.

## Introduction

Leishmaniases are vectorial parasitic diseases of mammals, including humans, caused by *Leishmania* species and present in all inhabited continents. It is estimated that 12 million people are infected, with an annual incidence of 2 million cases, and between ca. 350 million [1] and 3.4 billion people [2] living in areas at risk. It is considered the second most lethal parasitic disease, after malaria, visceralizing species being responsible of 20,000 to 40,000 human deaths per year [3]. In the last years a rise in human prevalence has been found, the disease extending to previously exempt areas. Control of infections relies on chemotherapy but these drugs have several shortcomings including high price, length of treatments and side effects such as toxicity and teratogenicity [4]. Moreover, resistance to the treatment of choice (antimonials) has been reported in endemic areas (e.g. India) [5] and new molecules are needed.

Allicin (2-Propene-1-sulfinothioic acid S-2-propenyl ester, diallyl thiosulfinate) and related compounds have been shown to inhibit the multiplication of neoplastic cell lines [6, 7]. The molecule has also shown antibacterial [8–10], antifungal [11, 12] and antiprotozoal activity [13–16]. More recently, antiproliferative activity of allicin against intracellular phases of *Leishmania* [17] and *in vivo* experimental infections with *L*.*infantum* [18] has been reported. Allicin easily diffuses across cell membranes and it has been described to react with thiol groups [19] and some other intracellular targets have also been incriminated (e.g. cysteine proteases, microtubules disruption) [16, 20–22] but the actual mechanism of action of allicin and the type of death induced are for the most part unknown. Allicin induces p53-mediated autophagy of Hep G2 human liver cancer cells [7] and apoptosis through caspase activation [23] and via Nrf2 [24]. Nevertheless, the closely related compound diallyl disulfide causes cell cycle arrest in the G_2_/M checkpoint in HCT-15 [25] and PC-3 [6] cell lines.

Information in unicellular eukaryotes is scarce although allicin seems to inhibit the expression of silent information regulator 2 (SIR2) gene (ortholog to mammalian SIRT1) [26] thus inhibiting the hyphae formation in the fungus *Candida* [27]; one of the metabolites of allicin, allyl alcohol, induces oxidative stress in this fungus. Preliminary transmission electron microscopy (TEM) studies of *Leishmania* promastigotes exposed to allicin showed that the most altered organelle was the mitochondrion [17]. The importance of this organelle in the energetic machinery of eukaryotic cells is critical in *Leishmania* and other trypanosomatids since they only have a large mitochondrion (ca. 12% of cellular volume) [28] and these organisms exhibit a scarce ability to survive and multiply in anaerobic environments [29].

Results presented indicate that diallyl thiosulfinate induces in *Leishmania* promastigotes a rapid elevation of cytosolic Ca^2+^ levels, high ROS generation, mitochondrial dysfunction with a collapse of the mitochondrial membrane potential (ΔΨm). These events lead to a bioenergetic catastrophe with fall of mitochondrial ATP production and cell necrosis with no evidence of apoptotic-like markers.

## Material and Methods

### Parasite culture and maintenance

The canine isolate of *L*.*infantum* (MCAN/ES/2001/UCM9) was employed in all the experimental procedures. Promastigotes were routinely cultured in 25 mL culture flasks at 27°C in RPMI 1640 modified medium (Lonza) supplemented with 10% heat-inactivated (30 min at 56°C) fetal bovine serum (TDI Laboratories, Madrid) and 100 U/mL of penicillin plus 100 μg/mL of streptomycin (BioWhittaker). Allicin was obtained as liquid Allisure from Allicin International Ltd. (Rye, East Sussex, UK) at a concentration of 5000 ppm and stored at -80°C until used.

### Measurement of reactive oxygen species (ROS) generation

Intracellular ROS levels were measured using the cell permeable probe H2DCFDA (2',7'-dichlorodihydrofluorescein diacetate, Molecular Probes). Experiments were carried out in triplicate following a modified protocol described by Fonseca-Silva et al. [30]. Briefly, 2 x 10^6^/mL mid-log phase promastigotes were incubated at 27°C for 3h in the absence or presence of increasing concentrations of allicin (15–120 μM). Parasites were washed in phosphate saline buffer (PBS) (Lonza), resuspended in 1 mL PBS (2 x 10^6^/mL) and incubated with 20 μM H2DCFDA for 20 min at 37°C, 5% CO_2_. Aliquots of 200 μL/well were transferred to a 96-well solid flat bottomed black microtiter plate (Costar, Corning) and fluorescence intensity was measured in a FLUOstar OPTIMA microplate reader (BMG Labtech) using excitation/emission wavelength of 500 nm/490 nm. Antimycin A (5 μM) (Sigma) was used as a positive control of ROS generation.

### Measurement of mitochondrial ROS generation

Superoxide anion (O_2_^-^) production was assayed fluorimetrically using the mitochondrial targeted probe MitoSox Red (Molecular Probes) as described previously by Carvalho et al. (2010) [31] with some modifications. Cells (10^7^ promastigotes/mL) were loaded with 1 μM MitoSox Red for 30 min at 27°C in HBSS (Ca/Mg) (Hank's Balanced Salt Solution with calcium and magnesium, Gibco). Parasites were washed in HBSS and then treated with allicin (15–120 μM) for 3h at 27°C. After treatment cells were washed and resuspended in HBSS (10^7^ promastigotes/mL). Aliquots of 200 μL/well were transferred to a 96-well solid black microtiter plate (Costar, Corning) and fluorescence intensity was recorded in a FLUOstar OPTIMA microplate reader (BMG Labtech) with an excitation wavelength of 510 nm and emission of 580 nm. Untreated cultures and cultures treated with 5 μM antimycin A (Sigma) were included as controls. Three experiments were carried out in triplicate.

### Measurement of free cytosolic calcium

Cytosolic Ca^2+^ in promastigotes was monitored using Fluo 4AM dye (Molecular Probes) using a modified protocol previously described [32]. Exponentially growing cultures were washed twice with the following loading buffer: 137 mM NaCl, 4 mM KCl, 1.5 mM KH_2_PO_4_, 8.5 mM Na_2_HPO_4_, 11 mM glucose, 2 mM CaCl_2_, 0.8 mM MgSO_4_ and 20 mM HEPES-NaOH, pH 7.4 (Sigma). A total of10^7^ cells/mL were preloaded with 5 μM Fluo 4AM for 60 min at 27°C in loading buffer. Pluronic F-127 (0.02%, Molecular Probes) was added to facilitate the dispersion of the nonpolar AM ester. Parasites were washed twice with loading buffer to allow complete intracellular de-esterification of the AM esters and cells were further incubated for 15 min at 27°C. Fluorescence was excited using a 485 nm filter and read through a 520 nm long-pass emission filter in a microplate reader (FLUOstar OPTIMA, BMG Labtech). To measure intracellular Ca^2+^ responses, baseline fluorescence was monitored before the addition of the different stimuli. Cells were treated with increasing concentrations of allicin (15–120 μM) and fluorescence intensity was recorded every 5 min for 1 h. Fluorescence intensity measurements were normalized [33]. Maximal fluorescence values were obtained by permeating cells with 0.5% Triton X-100 under saturated Ca^2+^ environment. Minimal fluorescence was measured after chelation of Ca^2+^ with 8 mM EGTA. Two independent experiments were carried out in triplicate.

### Measurement of mitochondrial transmembrane potential (ΔΨm)

Changes in the ΔΨm were analyzed by flow cytometry using the cationic lipophilic dye 5,5_,6,6_-tetrachloro-1,1_,3,3_-tetraethylbenzimidazole carbocyanide iodide (JC-1) (Molecular Probes) and the ratio between red/green fluorescence intensities (FL-2/FL-1; 590 nm/530 nm) [34]. Promastigotes treated for 3 h with allicin (15–120 μM, 27°C) were washed in PBS and resuspended (2 x 10^6^/mL) in 1 mL of PBS containing JC-1 dye at a final concentration of 6 μM. Parasites were incubated in the dark for 20 min at room temperature (RT) and washed twice in PBS to eliminate the non-internalized dye. Non-treated cells and cells treated with 100 μM of the mitochondrial uncoupler carbonyl cyanide 3-chlorophenylhydrazone (CCCP, Sigma) were included as controls. Measurements were performed using a FACScan flow cytometer and analyzed with CellQuest software (Becton Dickinson).

### Measurement of cellular ATP levels

Quantification of ATP levels was carried out using the CellTiter-Glo luminescent assay (Promega) [35]. Mid-log phase promastigotes (2 x 10^6^/mL) were incubated at 27°C in RPMI supplemented medium in the presence of 15, 30, 60, 90 and 120 μM allicin for 3 h. Untreated cultures and cultures treated with 20 mM sodium azide (Sigma) were included as controls. After the drug exposure period, a 30 μL aliquot of the parasite suspension was transferred to 96-well solid white flat bottomed microtiter plates (Costar, Corning) and an equal volume of CellTiter-Glo was added to each well. Plates were incubated in the dark for 10 min at RT and luminescence was measured using a FLUOstar Omega microplate reader (BMG Labtech). Three independent experiments were carried out in triplicate.

### Determination of plasma membrane integrity

Cell membrane permeabilization was determined using the SYTOX Green nucleic acid stain (Molecular Probes) [36] with modifications. Mid-log phase promastigotes were washed twice in HBSS and parasites (2 x 10^6^ promastigotes/mL) were incubated (15 min, 27°C) in the dark with 2 μM SYTOX Green. Cells were incubated in the presence of increasing concentrations of allicin (0, 15, 30, 60, 90 and 120 μM) for 3 h, 27°C. An aliquot of the parasite suspension (200μL/well) was transferred to a 96-well solid black microtiter plate (Costar, Corning) and fluorescence intensity was measured in a FLUOstar OPTIMA microplate reader (BMG Labtech) with excitation and emission wavelengths of 520 nm and 500 nm, respectively. Control for maximum fluorescence (100% membrane permeabilization) was obtained by the addition of 0.5% Triton X-100. Three independent experiments were carried out in triplicate.

### Determination of trypanothione reductase (TryR) activity in parasite lysates

Assessment of TryR activity was carried out using the colorimetric method described [37]. *Leishmania* logarithmic promastigotes (2 x 10^6^/mL; 50 mL) were grown in 75 cm^2^ culture flasks and exposed to different allicin concentrations (30, 60 and 120 μM) for 3 h at 27°C. Untreated cultures were included as negative controls. The tricyclic neuroleptic drug clomipramine HCl (Sigma) has previously been reported to selectively inhibit TryR [38]. Positive control cultures treated with the TryR inhibitor, clomipramine HCl (Sigma), 10 μM (3h, 27°C) were also included in the experiment. Cells were washed twice in PBS and cultures were concentrated (2 x 10^7^/mL; 1mL/eppendorf). Samples were centrifuged (7826 xg, 5 min, RT) and supernatants were discarded. Pellets were incubated for 15 min at RT with 1 mL of lysis buffer consisting of 1 mM EDTA, 40 mM HEPES, 50 mM Tris-HCl (pH 7.5), and 2% v/v Triton X-100 (Sigma). Prior to lysis, buffer was supplemented with 1 mM protease inhibitor phenylmethanesulfonyl fluoride (PMSF, Sigma). Stock solutions and further dilutions of NADPH (Sigma), T[S]_2_ (Bachem) and DTNB (Sigma) were stored and prepared as described by van den Bogaart et al. (2014) [37]. To determine TryR activity, an aliquot (375 μL) of the parasite lysate was transferred into a new microcentrifuge tube, followed by the sequential addition of NADPH (125 μL/sample), T[S]_2_ (375 μL/sample) and DTNB (125 μL/sample) yielding final concentrations of 200, 75 and 100 μM, respectively. A blank was included for each sample consisting of the same reaction mixture without the substrate. T[S]_2_ was replaced by an equal volume of 0.05M Tris buffer (pH 7.5). Samples were incubated at 27°C protected from light and absorbance (λ = 412 nm) was determined for 90 min every 15 min in a UV Mini 1240 spectrophotometer (Shimadzu). Blank values were subtracted from all the samples.

### Determination of non-protein thiol levels: GSH, T[SH]_2_ and Cys

L-cysteine (Cys) (97%), glutathione (GSH) (99%), 2,3-dimercapto-1-propanol (DMP) (98%), methanesulfonic acid (MSA) (99.5%), tris (2-carboxyethyl)phosphine hydrochloride (TCEP-HCl) (98%), diethylenetriaminepentaacetic acid (DTPA) (99%) were purchased from Sigma. Trypanothione (T[SH]2) (>99%) was obtained from Bachem AG, monobromobimane (mBBr) was from Toronto Research Chemicals.Acetonitrile (MeCN) and methanol (MeOH) (HPLC-grade) were provided by Scharlab and trifluoroacetic acid (TFA) (HPLC-grade, 99.5%) was from Apollo Scientific. Analytical grade reagents: 3-[4-(2-hydroxyethyl)-1-piperazinyl) propane sulfonic acid (HEPPS) (99%) was from AppliChem and sodium hydroxide (NaOH) was from Thermo-Fisher Scientific. All solutions used in the HPLC were passed through a 0.45 μm nylon filter before use. Exponentially growing promastigotes (2 x 10^6^/mL; 50–100 mL) were harvested in triplicate and treated for 24 h with increasing allicin concentrations at 27°C. Cell suspensions were washed twice in PBS and frozen (2 x 10^7^ cells; 50 μL PBS) at -80°C until the time of analyses. GSH, Cys, and T[SH]_2_ stock solutions were prepared in Milli-Q water at a concentration of 8 mM, 8 mM and 15 mM, respectively. DMP stock solution was prepared in Milli-Q water at a concentration of 8 mM. A stock solution of mBBr was prepared in acetonitrile at a concentration of 50 mM and 20 mM TCEP solution was made in 200 mM HEPPS buffer (pH 8.2). All solutions were aliquoted and stored at -20°C in the dark until the time of analysis. Appropriate aliquots of Cys, GSH and T(SH)_2_ stocks were mixed and diluted with extraction solution (6.3 mM DTPA with 0.1% TFA) to construct a calibration curve of each thiol with concentration ranging between 0.05 and 6.2 nmol/mL. The internal standard, DMP, was prepared at a final concentration of 0.75 nmol/mL. The extraction of thiols was performed in acid media using as extraction solvent 50 μL of 6.3 mM DTPA (0.1% TFA) that was added to microcentrifuge tubes containing the pellets resuspended in PBS. After vortex mixing, *Leishmania* extracts were immediately frozen in liquid N_2_ and thawed three times to fully release cellular content. The supernatant was collected by centrifugation (13,000 rpm, 10 min) and subsequently derivatized. The derivatization procedure followed was based on the method described [39]. Firstly, 10 μL of 20 mM TCEP and 246 μL of 200 mM HEPPS buffer (6.3 mM DTPA, pH 8.2) were mixed to obtain a sulfur reducing solution that was subsequently added to 100 μL aliquots of *Leishmania* sample extracts or standards; 4 μL of 75 μmol/L DMP was also added as an internal standard. Then, the mixtures were incubated at 45°C in a water bath for 10 min to guarantee the reduced state of thiols before mBBr derivatization. For derivatization of thiols, 10 μL of 50 mM of mBBr was added to the vials containing samples or standards and incubated in the dark for 30 min at 45°C in a water bath. Finally, 100 μL of 1 M MSA were added to stop reaction and derivatized samples were analyzed by HPLC-DAD/FLD [High-performance liquid chromatography (HPLC) equipped with both diode-array detector (DAD) and a fluorescence detector (FLD)]. Chromatographic separation of the thiols was performed on a SynergiTM Hydro-RP (250 mm × 4.6 mm, 4 μm) HPLC column from Phenomenex (Torrance, CA, USA). A gradient program was used with the mobile phase, combining solvent A (Milli-Q water with 0.1% TFA) and solvent B (MeCN with 0.1% TFA) as follows: 10% B (5 min), 20.6% B (6.7 min), 31.1% B (13.6 min) and 100% B (5 min). The column was equilibrated with 10% of solvent B for a total of 8 min before next injection. Analyses were performed at a flow rate of 1.0 mL/min and the column temperature was kept at 40°C. The injection volume was 100 μL and all the compounds elute within 30 min. The DAD detector was set at both 290 and 380 nm and the excitation and emission wavelengths of the FLD detector were set at 380 nm and 470 nm, respectively. Quantification was performed using internal calibration and peak area measurements. Multiple analyses were performed using blanks and standards to determine detection limits, response linearity and reproducibility of the protocol.

### Annexin V binding and propidium iodide staining

Changes in the transbilayer arrangement of phospholipids [40] in *Leishmania* promastigotes was analyzed using the Annexin V-FLUOS Staining kit (Roche). Cells (2 x 10^6^/mL) were incubated at 27°C in the presence of allicin (30–120 μM) for 3, 12, 24 and 48 h. Cells were washed twice in cold PBS and the resulting pellet resuspended in 100 μL of HEPES buffer containing Annexin-V and PI (2 μL of each). Samples were incubated at RT in the dark for 15 min and analyzed by flow cytometry (FACScan, Becton Dickinson) with CellQUEST software. Cultures incubated at 45°C overnight were used as positive death controls.

### DNA fragmentation assay by agarose gel electrophoresis

Qualitative analysis of DNA fragmentation was performed by agarose gel electrophoresis of total genomic DNA extracted from untreated and allicin treated promastigotes. *Leishmania* promastigotes were exposed to allicin (30 μM and 60 μM) for 24, 48 and 72h. After drug exposure cells were washed twice in sterile PBS (1000 *x*g, 10 min, RT) and the total DNA was extracted from the cell pellet (10^8^ promastigotes) using the Apoptotic DNA ladder kit (Roche) following manufacturer’s protocol. The DNA was quantified at 260/280 nm using a NanoDrop ND-1000 spectrophotometer. The genomic DNA (5 μg) was run on a 2% agarose gel containing SYBR Safe DNA gel stain for 1 h at 100 V and visualized under UV light using the DNA Molecular Weight marker XIV (Roche).

### Detection of DNA fragmentation in situ and cell cycle analysis

TUNEL (dUTP nick-end labeling) was performed using an APO-BrdU TUNEL Assay Kit (Invitrogen). Briefly, promastigotes (2 x 10^6^/mL) were treated with allicin for 48 and 72 h and washed twice with cold PBS. Parasites were fixed in 1% paraformaldehyde (4°C, 15 min), washed and incubated at -20°C overnight in 70% ethanol. After washing, cells were incubated (60 min, 37°C, dark) in DNA-labeling solution to allow TdT-mediated DNA-BrdU binding. Cells were washed twice in rinsing buffer and incubated (30 min, RT, darkness) with Alexa Fluor 488 conjugated anti-BrdU antibody. Prior to flow cytometric analyses (FACScan, CellQUEST software). PI/RNase A staining solution was added to examine cellular DNA content and study cell cycle progression.

### Transmission electron microscopy (TEM)

Cells were fixed with 2% glutaraldehyde in 0.1 M sodium cacodylate buffer, pH 7.4, for 90 minutes at 4°C. After washing (cacodylate buffer + 4.5% sacharose) promastigotes were post-fixed with 1% osmium tetroxide for 1 h in the same buffer. Samples were dehydrated with ethanol/water serial dilutions, stained with 1% uranyl acetate in 70% ethanol, 45 min, 4°C and included in epoxy resin EPON 812. After polymerization (24 h, 45 1°C and 48 h at 60°C), ultrathin sections (ca. 50–60 nm) were obtained with a Leica EM UC6 ultramicrotome. Sections were collected in copper-rhodium grid 300 mesh and stained with 1% uranyl acetate and lead citrate. Sections were studied with a TEM/STEM Philips Tecnai 12 microscope and images were obtained with digital camera or plates.

### Statistical analysis

Statistical analysis and graphs were performed with GraphPad Prism 5 software. Statistical significance of differences was determined by one-way and two-way analysis of variance (ANOVA) and Bonferroni post-test. Differences were considered significant at a *p* value of < 0.05.

## Results

### Allicin induces cytosolic and mitochondrial ROS production in promastigotes of *Leishmania*

**Fig 1:**
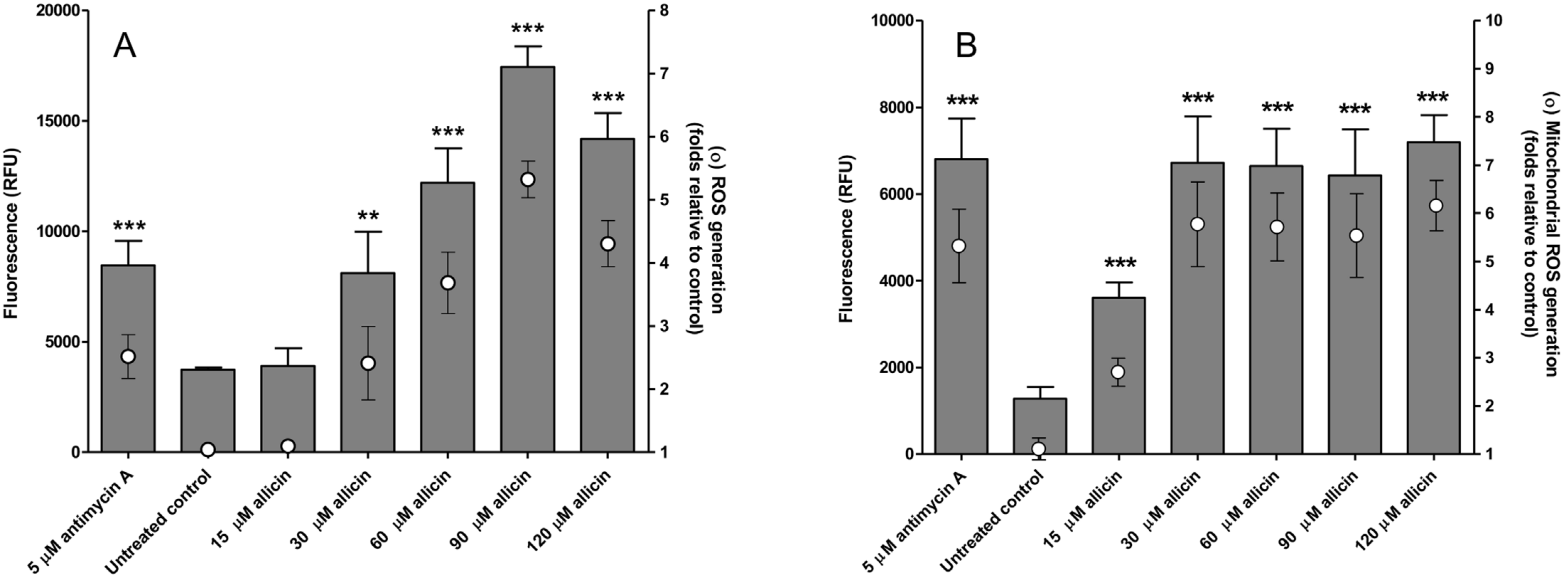
Allicin induces ROS generation in *Leishmania* promastigotes in a concentration-dependent manner. A, intracellular generation of ROS estimated with the H2DCFDA probe in promastigotes treated for 3h with 15–120 μM allicin, untreated control cultures and positive control exposed to 5 μM antimycin A. B, effect of allicin on mitochondrial ROS generation by MitoSox Red in promastigotes treated for 3 h. Figure shows the results in Relative Fluorescence Units (RFU) and folds relative to untreated control cultures. Results shown correspond to means ± standard deviation (S.D.) of three experiments in triplicate and asterisks represent significant differences related to untreated cultures (***: *p*<0.0001 **: *p*<0.001).

Allicin triggered the intracellular levels of hydroxyl radicals, hydrogen peroxide and peroxynitrites (Fig 1A) in the promastigote stage of *Leishmania* after 3 h exposition. Intracellular ROS generation was concentration dependent and reached over 5-fold production in the presence of 90 μM allicin. Exposition of promastigotes to the estimated EC_50_ value (30 μM) elicited a ROS production similar to the value reached by the positive control (5 μM antimycin A). Similarly, allicin effectively induced a strong elevation of superoxide production in mitochondria of treated cells (Fig 1B) as determined by the mitochondrial targeted probe (MitoSox Red). The induction was notable even with moderate allicin concentrations and a 6-fold increase was found with a concentration of 30 μM. It should be indicated that this increase, in the region of the levels reached by the positively treated promastigotes (5 μM antimycin), was maintained with higher allicin concentrations (60–120 μM).

### Allicin reduced moderately trypanothione reductase (TryR) activity and increased non-protein thiol levels in *Leishmania*

**Fig 2:**
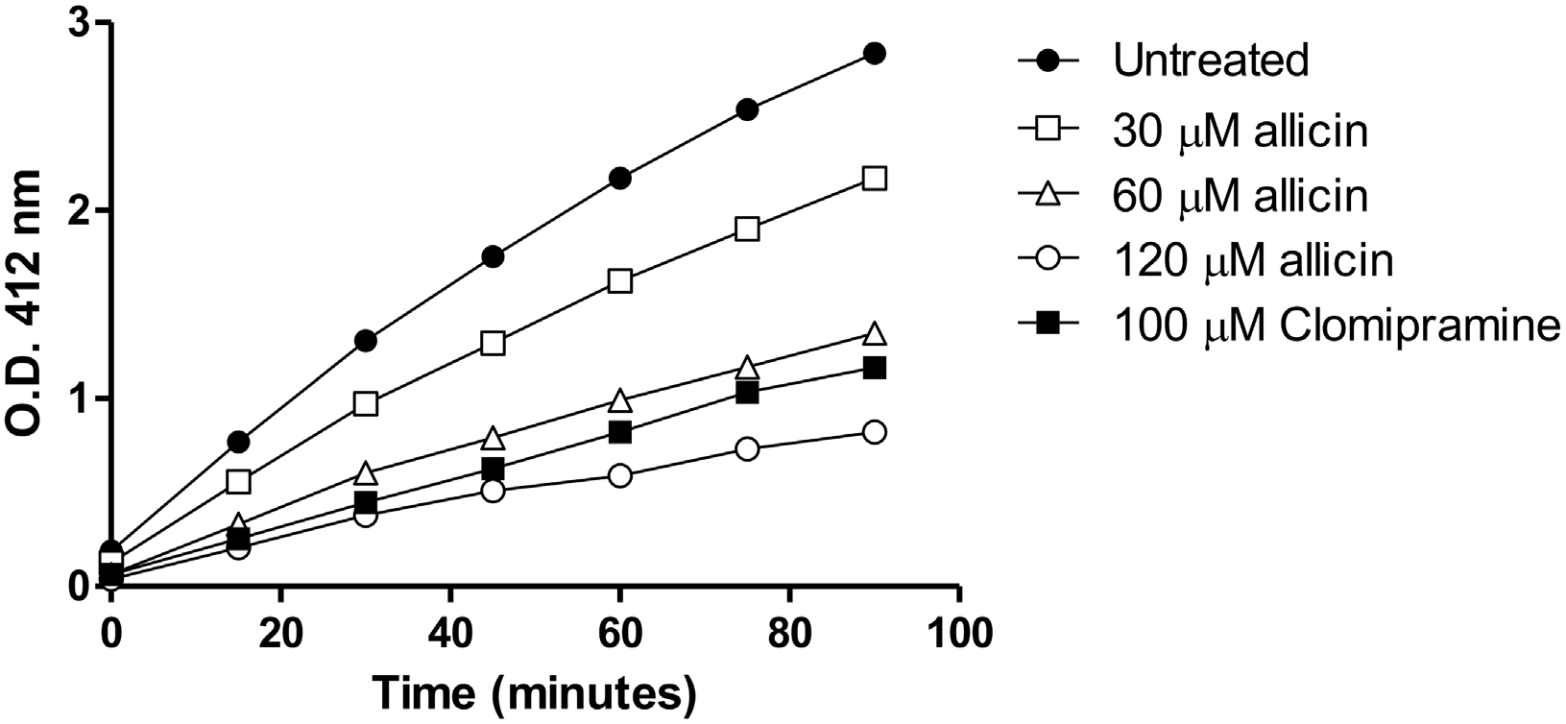
Inhibition of TryR activity of *Leishmania* by allicin. Promastigotes treated for 3 h with varying concentrations of allicin (30, 60 and 120 μM) were lysed and the enzyme activity was determined by the sequential addition of 200μM NADPH, 75 μM trypanothione and 100 μM DTNB. Thionitrobenzoate ion (TNB^2-^) produced was proportional to TryR activity and was quantified spectrophotometrically (412 nm) at different time points. Untreated control cultures and cultures treated with the TryR inhibitor clomipramine (100 μM) were included. Data shown correspond to the mean values of a representative experiment, from the two executed, carried out in duplicate.

**Fig 3:**
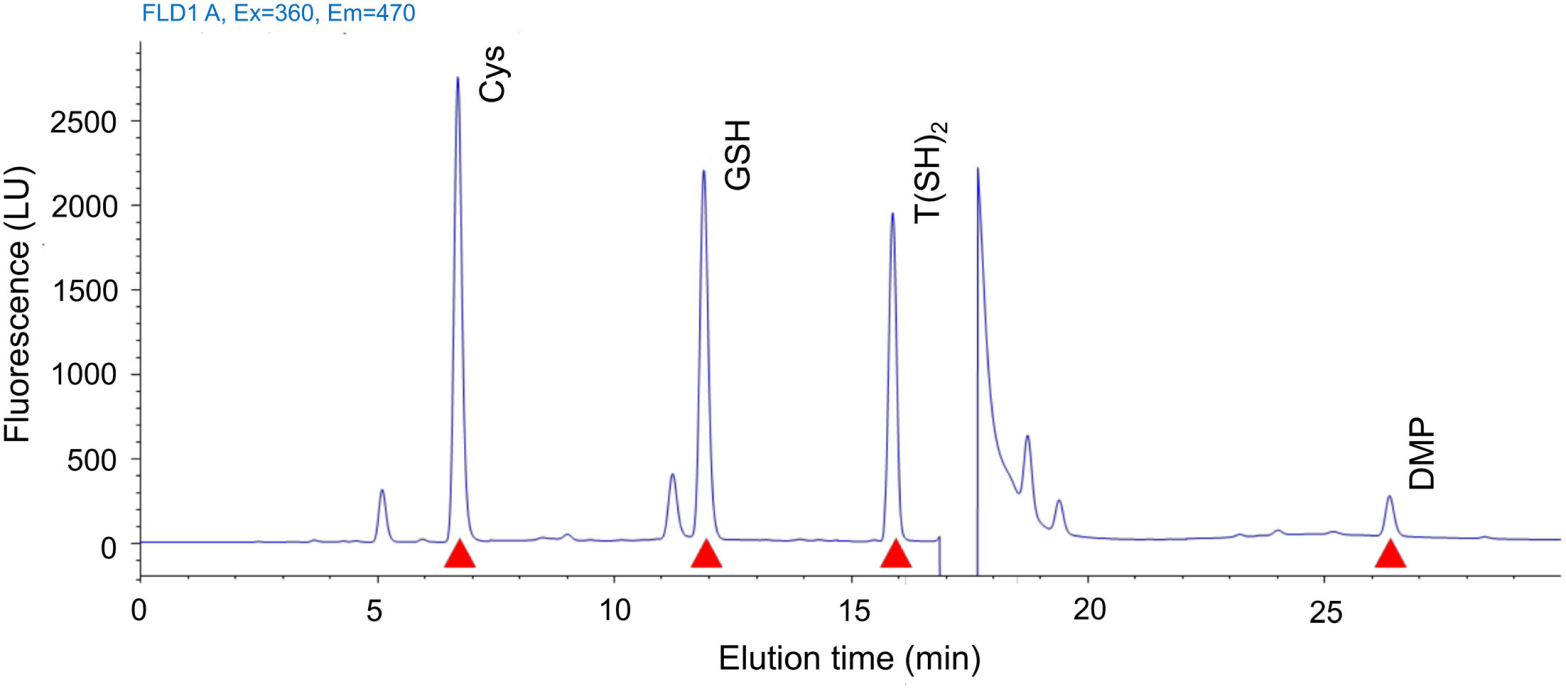
HPLC elution profile of MBBr derivatized thiols. Standard solution of Cys, GSH and T[SH]_2_ prepared at 3 nmol/mL; 0.075 nmol/L of DMP was added as an internal standard.

Trypanothione reductase (TryR) plays a fundamental role in the antioxidant defense of the cells through the reduction of trypanothione, the unique thiol present in Kinetoplastida including *Leishmania*. Exposure of *Leishmania* promastigotes to allicin for 3 h provoked a dose-dependent moderate reduction of TryR activity in parasite lysates of treated cells. Residual activity in the presence of the lowest allicin concentration (30 μM) reached a 66.67% of that found in untreated control cultures; 120 μM allicin reduced the TryR activity by 80%, inhibition higher than that induced by the specific inhibitor clomipramine. Remaining TryR present in the leishmanial lysates displayed standard enzymatic kinetics (Fig 2). Non-protein cellular thiols of *Leishmania* were separated, identified and quantified using HPLC-DAD-FLD. This approach was modified to improve reproducibility and sensitivity. The optimized separation was performed in gradient elution mode that yielded resolution (Rs) higher than 2.0 and retention times (rt) of 6.70, 11.94, 15.90 and 26.43 min for Cys, GSH, T[SH]_2_ and DMP (2,3 –dimercapto-1-propanol) respectively. The rt were reproducible between runs. Fig 3 shows a representative chromatogram of a standard mixture of the thiols. Thiols were quantified using an internal standard (DMP) calibration model using an eight-point calibration curve, which covered three-log concentration range (0.05–6.2 nmol/mL) for the three monothiols Cys, GSH and T(SH)_2_. The *r*^2^ value for standard curves was >0.999 for all thiol compounds tested. No clear figure emerged, in the experiments carried out, although the tendency suggested that non-protein thiol levels increased with allicin treatment at low concentrations (≤ 60 μM) although T[SH]_2_ levels remained ca. 1.0 and 2.0 nmol/10^8^ cells irrespective of the allicin concentration; only with 100 and 120 μM allicin, when TryR activity was reduced over 80% (see above), the levels of the thiol fell. By its part, the levels of GSH showed a pattern of increased levels with low allicin concentrations (< 30–50 μM), up to 2.5–3.0 nmol/10^8^ cells, followed by reduced levels with higher concentrations of the drug. Levels of cysteine were more variable between experiments although in all determinations there was a steady increase with higher drug concentrations (Table 1).

**Table 1:** Levels of non-protein thiols (cysteine, trypanothione and glutathione) in promastigotes of *L*.*infantum* treated with allicin (10–120 μM)*.

Allicin (μM)	nmol/10^8^ cells					
	Cys		GSH		T[SH]_2_	
	mean	S.D.	mean	S.D.	mean	S.D.
0	0.723	0.194	0.723	0.126	1.162	0.130
10	0.914	0.214	0.914	0.146	1.591	0.192
20	1.179	0.684	**1.179**	**0.211**	1.998	0.231
30	**1.448**	**0.801**	**1.448**	**0.194**	**2.235**	**0.135**
40	1.371	0.962	**1.371**	**0.154**	2.162	0.173
50	**1.561**	**0.711**	**1.561**	**0.184**	**2.365**	**0.112**
60	**1.869**	**0.909**	**1.869**	**0.173**	**2.217**	**0.206**
80	**1.675**	**0.849**	**1.675**	**0.166**	**3.076**	**0.255**
100	**1.656**	**1.079**	**1.656**	**0.138**	**0.655**	**0.069**
120	**1.771**	**0.762**	**1.771**	**0.100**	**0.216**	**0.012**

* Bold values are significantly different (*p*<0.05—*p*<0.001) to the untreated control within the same column.

### Allicin triggers cytosolic Ca^2+^ in *Leishmania* promastigotes

**Fig 4:**
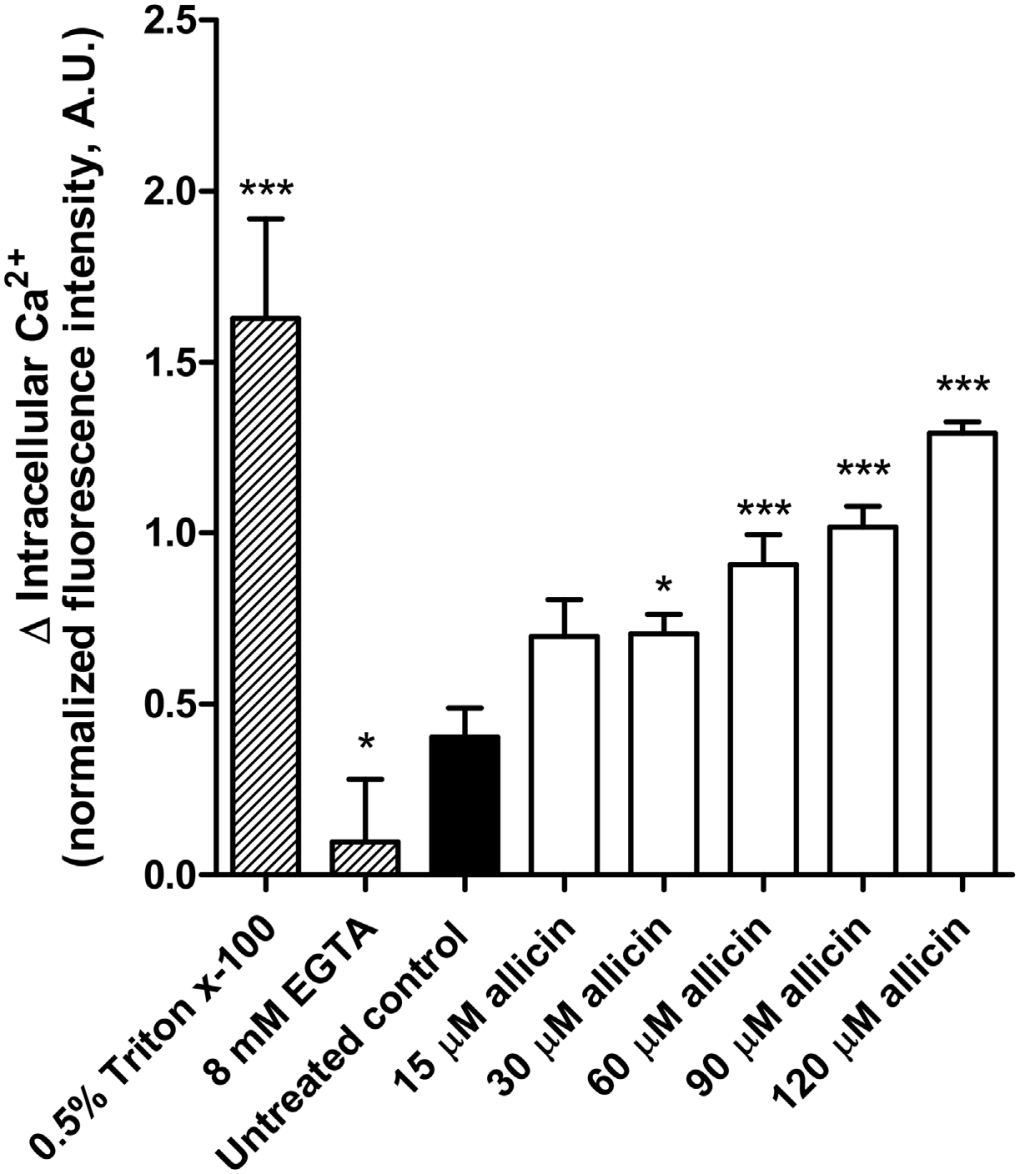
Intracellular Ca^2+^ in *Leishmania* increases in response to allicin stress. *Leishmania* promastigotes were treated with allicin (15–120 μM) for 20 min. Calcium levels were determined by Fluo 4AM preloading of cells (60 min) in loading buffer with 0.02% Pluronic F-127. Maximal fluorescence was obtained with cells treated with 0.5% Triton X-100 and minimal after chelation with 8 mM EGTA. Data presented corresponds to the average increase of normalized fluorescence intensity of two independent experiments in triplicate (mean ± S.D.). Asterisks represent significant differences compared to untreated control promastigotes (*: *p*<0.05; ***: *p*<0.001).

The cytosolic levels of calcium in *L*.*infantum* promastigotes treated with increasing concentrations of allicin (15–120 μM) were determined at different times (0–60 min). For comparative purposes results obtained were normalized using as baseline the fluorescence before adding stimuli. Addition of allicin induced a rapid increase of cytosolic Ca^2+^ in a dose-dependent manner for a given post treatment time. Fig 4 shows the results obtained after 20 min. Chelation with 8 mM EGTA inhibited this increase whereas the permeabilization of the cell membrane of *Leishmania* with 0.5% Triton X-100, in a saturated Ca^2+^ environment, showed the maximal cytosolic concentration. The increase of calcium was very rapid and after 20 min cells treated with the EC_50_ concentration (30 μM allicin) reached ca. 43% of the maximal value obtained with the positive control.

### Allicin alters the mitochondrial membrane potential of *Leishmania*

**Fig 5:**
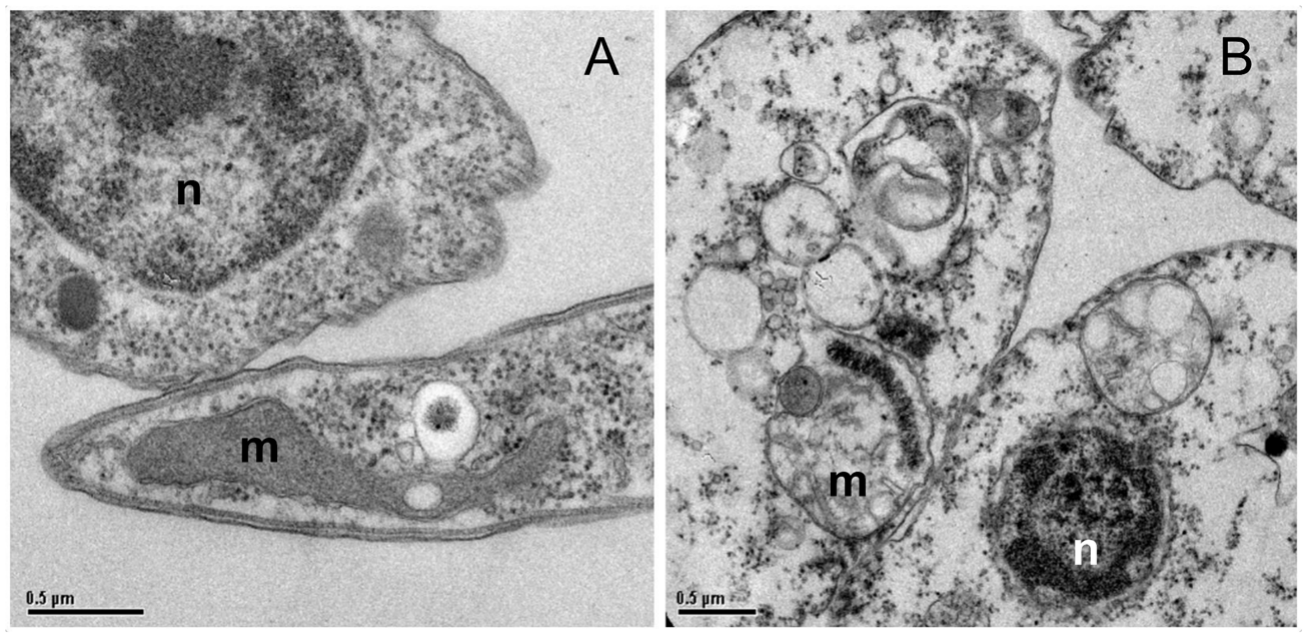
Electron micrographs of *Leishmania* promastigotes exposed to allicin. Untreated (A) and treated (24 h exposure to 90 μM allicin) *Leishmania* promastigotes (B). m: mitochondrion, n: nucleus. Bar = 0.5 μm.

**Fig 6:**
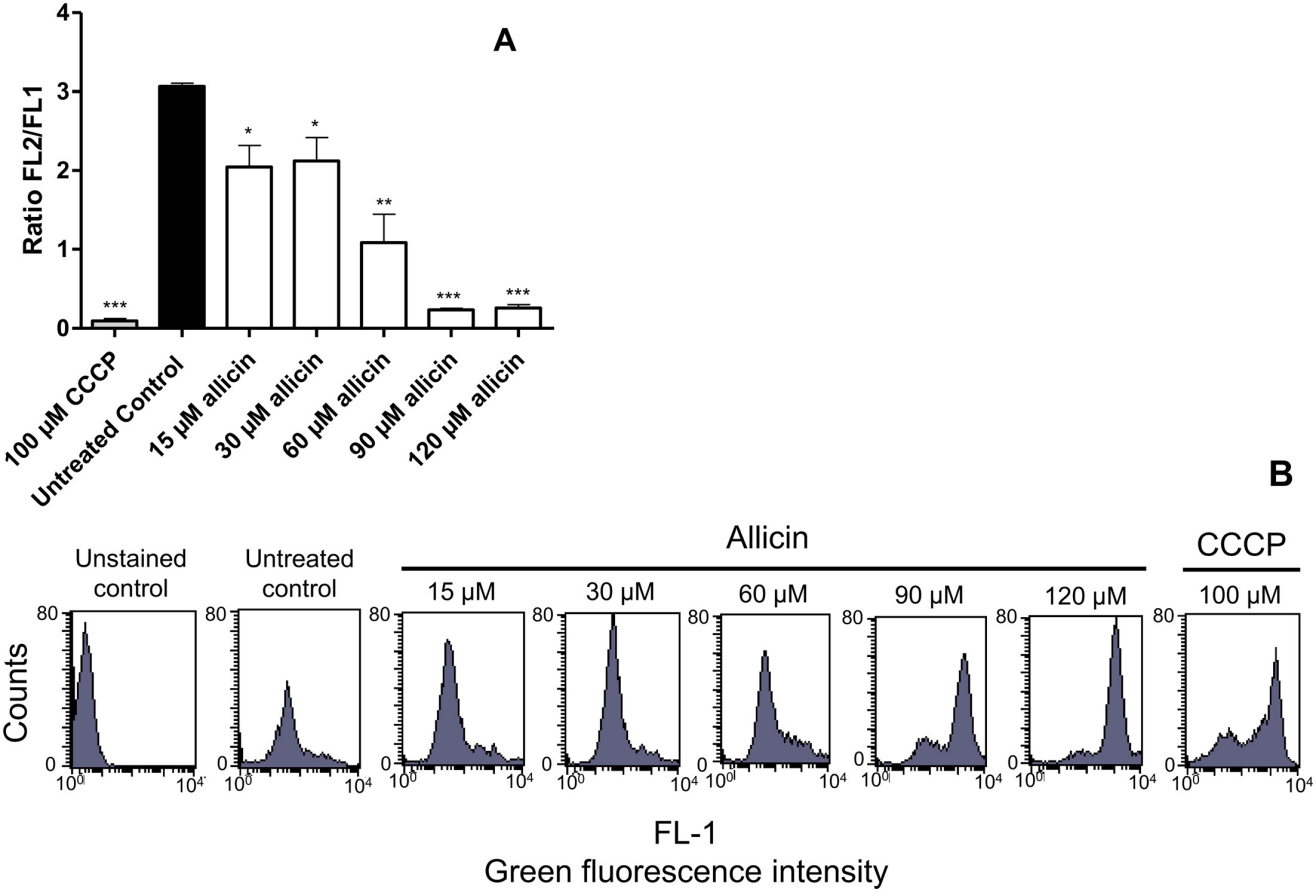
Allicin provokes the loss of ΔΨm in *L*.*infantum* promastigotes. Cells were exposed for 3 h to 15–120 μM allicin and fluctuations of ΔΨm were determined by flow cytometry of cells preloaded with JC-1. Fluorescence intensity was determined by FACS analysis (green, FL-1 and red, FL-2). Untreated and positive (100 μM CCCP treated cells, 3 h) cultures were included. A: Effect of allicin on FL-2/FL-1 ratio. Fluorescence intensity was determined by FACS analysis. (FL-2: J-aggregates and in FL-1: JC-1 monomers). Untreated control cells and promastigotes exposed to the mitochondrial membrane uncoupler CCCP were included as controls. Data represents the mean ± S.D. of three determinations. Asterisks represent significant differences to the untreated control cultures (*: *p*<0.05; **: *p*<0.005; ***: *p*<0.001). B: Representative FACS analysis of monomeric JC-1 (green fluorescence intensity, FL-1) levels in promastigotes of *L*.*infantum* treated with allicin, untreated or subjected to the mitochondrial uncoupler CCCP (100 μM).

TEM studies showed that allicin induced a range of ultrastructural alterations in *Leishmania* promastigotes including generalized vacuolization. Most prominent lesions were observed in mitochondrion and kinetoplast with notable deformation, swelling of the organelle and loss of internal integrity (Fig 5). Evaluation of mitochondrial transmembrane potential (ΔΨm) of *L*.*infantum* promastigotes treated with allicin was carried out using the cationic dye JC-1 and its selective accumulation in mitochondria inversely related to ΔΨm. Net negative charge of mitochondrial membrane is a characteristic of healthy cells thus allowing the concentration of the cationic dye. JC-1 aggregates emit red fluorescence at higher potential whereas with membrane potentials below 140 mV remains as a monomer within the cytoplasm emitting green fluorescence. The potential can be altered by some intracellular events (e.g. ROS production) in *Leishmania* and our results showed an increase in mitochondrial ROS production in the presence of allicin, even at low micromolar concentrations. To asses changes in the ΔΨm in the presence of allicin it was considered relevant to determine the ratio between red fluorescence (590 nm or FL-2) and green fluorescence (530 nm or FL-1). Fluorescence intensity as determined by FACS analysis (Fig 6A) showed that the FL-2/FL-1 ratio (red/green fluorescence ratio) was significantly affected, in our experimental conditions, in a dose-related manner since only 15–30 μM allicin induced a 23–30% reduction of the ratio and this value was below 90% with allicin concentrations >90 μM. Furthermore, the depolarization of the mitochondria evidenced by the ΔΨm fall was supported by the shift of the *Leishmania* population towards the right in the FL-1 channel (green fluorescence) as observed by FACS analysis (Fig 6B).

### Cellular levels of ATP in *Leishmania* promastigotes exposed to allicin are reduced without significant cell membrane damage

**Fig 7:**
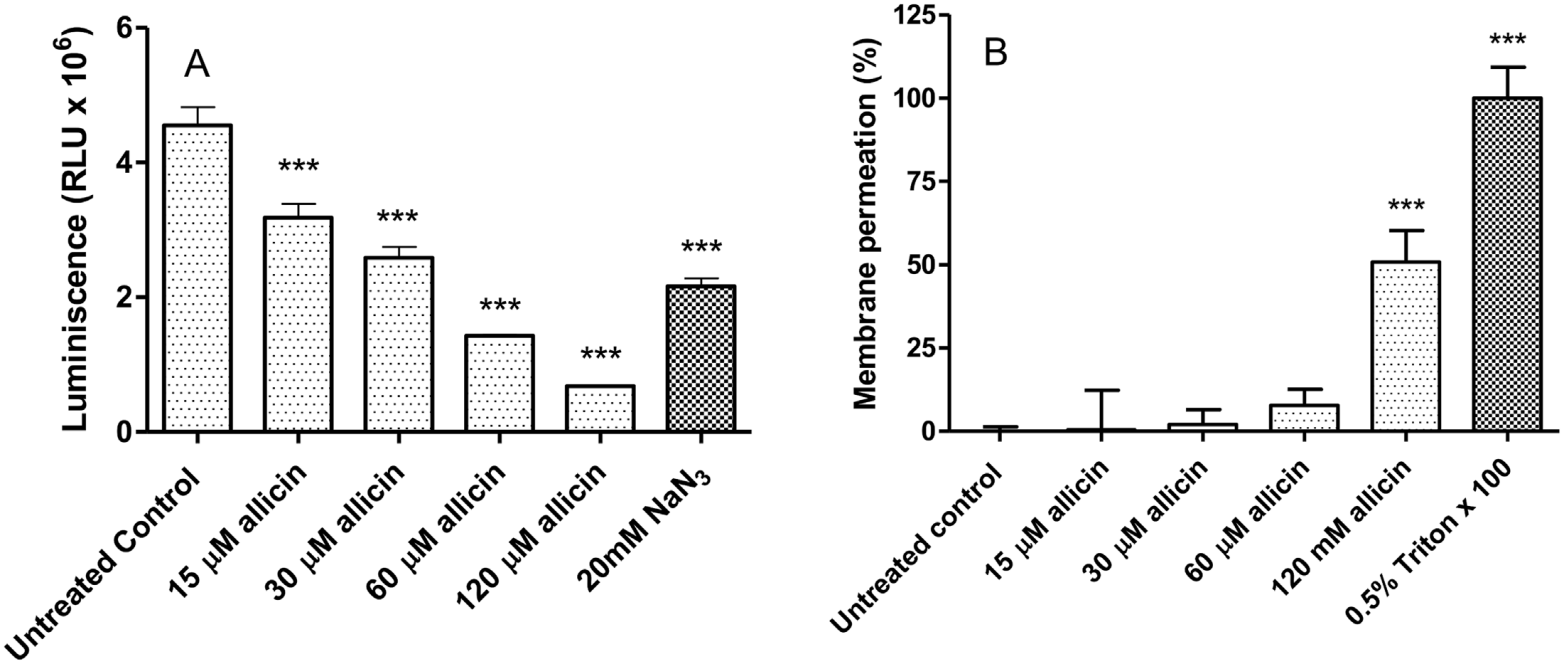
Allicin induces the fall of ATP production by *Leishmania* without loss of cell membrane integrity. A: Effect of allicin (15–120 μM, 3 h) on ATP levels of *L*.*infantum* promastigotes determined by the CellTiter-Glo luminescent assay. Untreated control cells and cells treated with 20 mM sodium azide as inhibitor of mitochondrial oxidative generation of ATP were included. Data presented corresponds to one representative experiment of the three independent experiments in triplicate carried out. B: Effect of allicin on membrane permeabilization. Promastigotes of *Leishmania* were incubated with allicin (15–120 μM) for 3 h and the permeability to SYTOX Green nucleic acid stain determined. Untreated and positive samples exposed to 0.5% Triton X-100 were included. Data represents means ± S.D. of three independent experiments carried out in triplicate. Asterisks represent significant differences to the untreated control cultures (***: *p*<0.001).

Allicin induced a dose-dependent fall of ATP levels in *Leishmania*, since exposure to 30 μM allicin (3 h) reduced ATP levels over 40% (2.59 ± 0.16 Relative Luminescence Units, RLU, in 30 μM allicin-treated promastigotes versus 4.55 ± 0.27 RLU in untreated control cultures). The decrease of ATP levels caused by 60 μM allicin was significantly higher (*p*<0.001) than the fall provoked by 20 mM sodium cyanide (Fig 7A). Exposition to allicin (≥ 90 μM) induced altered permeability of plasma membrane of promastigotes of *Leishmania* (*p*<0.001); 120 μM allicin yielded ca. 50% increased permeability from that obtained with the positive control (0.5% Triton X-100) at least as assessed by SYTOX Green internalization (Fig 7B). Interestingly, low concentrations of diallyl thiosulfinate, and in particular in the range of EC_50_ values for this parasite stage (ca. 30 μM), did not result in any significant alteration of the cell membrane. These results point towards the diminished intracellular ATP generation observed in treated cells (<60 μM; i.e. drug concentrations that did not induce a notable damage of the plasma membrane) was probably due to allicin.

### Time course effects of allicin on *Leishmania* promastigotes

**Fig 8:**
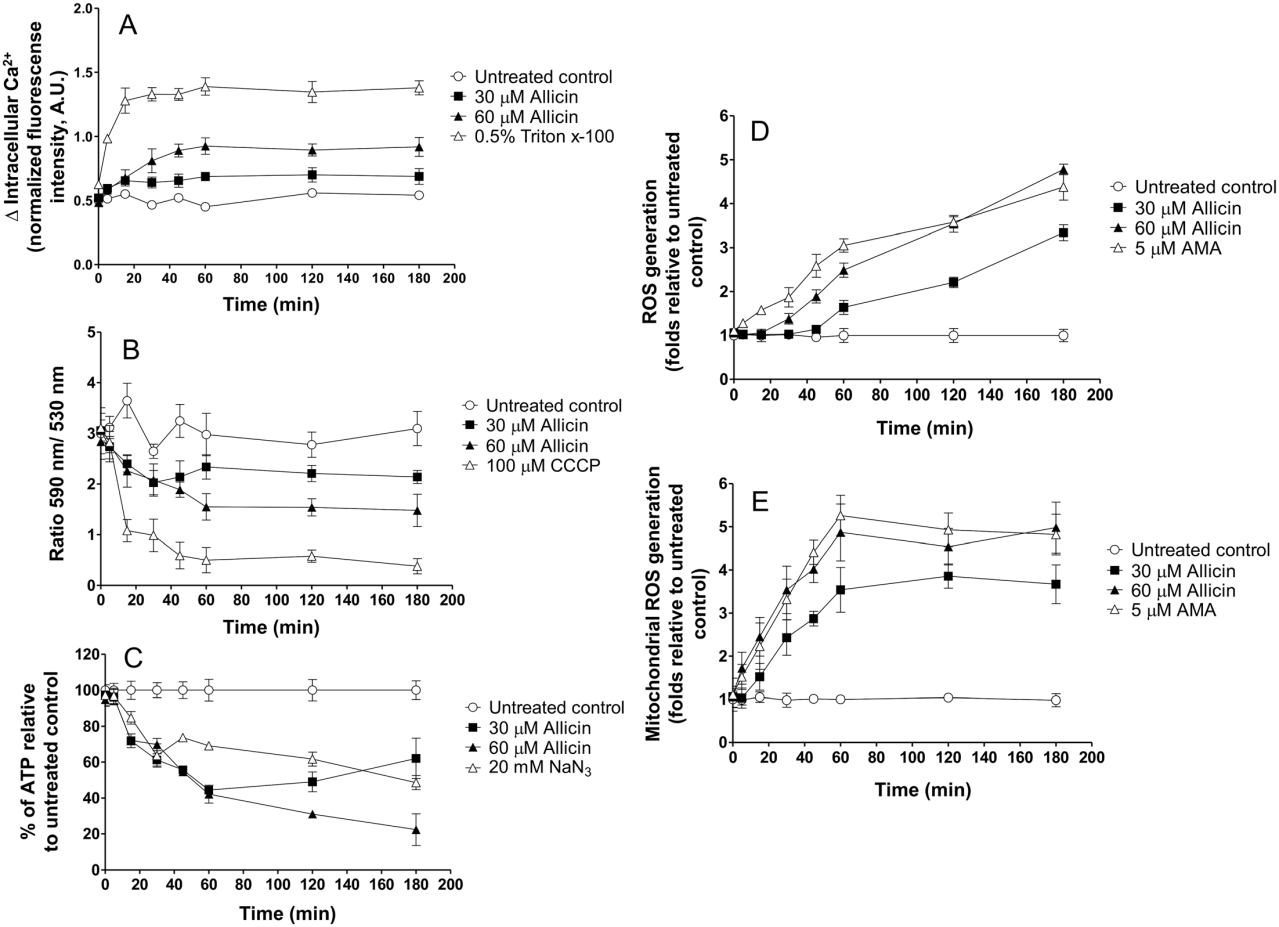
Time-course effect of allicin on ROS levels, intracellular Ca^2+^, mitochondrial transmembrane potential and ATP production. *Leishmania* promastigotes were exposed to allicin (30 μM and 60 μM). Intracellular Ca^2+^ levels (A), transmembrane mitochondrial potential (B), ATP production (C), cytosolic ROS (D) and mitochondrial ROS levels (E) were determined at 0, 5, 15, 30, 45, 60, 120 and 180 minutes post-treatment. Methods employed for each determination were those given in Material and Methods. Results are the mean ± S.D. of three determinations. Asterisks represent statistically significant differences with untreated controls (*: *p*<0.05; ** *p*<0.001).

Previous experiments provided conclusive evidence of the effect of allicin on increased Ca^2+^, ROS generation, both cytosolic and mitochondrial, and a fall of mitochondrial membrane potential and ATP production. These findings suggested that mitochondrion could be the target of diallyl thiosulfinate. However all experiments, with the exception of calcium levels determination, were performed at a fixed incubation time of 3 h. Moreover, mitochondrial alterations were observed after 24 h incubation with allicin concentrations (90 μM) well over the estimated EC_50_ value (ca. 30 μM). Therefore a time-course study was carried out to determine the sequence of events and thus the potential primary mechanism of action of allicin (Fig 8).

Results obtained in the time-course study confirmed the previous findings with fixed time experiments (3 h). Allicin elicited a time- and concentration-related alteration of all parameters determined when compared to untreated *Leishmania* cells. Thus, after 1 h incubation with 30 μM allicin, the estimated EC_50_, cytosolic ROS increased ca. 50% (Fig 8D) and superoxide levels in mitochondria were elevated over three times the basal values (Fig 8E). In a similar way intracellular calcium levels reached after 1 h > 40% increase over untreated cells (Fig 8A). By its part the fall of ΔΨm (Fig 8B) and ATP production (Fig 8C) represented between a 35–40% of the values found in control cells. Alterations were increased with higher allicin concentrations (60 μM). With the exception of ATP production and cytosolic ROS maximum effects were observed after 1 h treatment and remained almost constant for the entire duration of the experiments. Effect of allicin was rapid since treatment of promastigotes with 30 μM and μM 60 allicin induced significant increases (*p*<0.001) of cytosolic calcium after 15 min and 5 min, respectively. This elevation was accompanied by a clear fall of mitochondrial membrane potential, ATP production and mitochondrial ROS generation with both allicin concentrations tested after 15 min (*p*< 0.001). By its part cytosolic ROS showed a steady increase along the experiment and their levels were significantly higher from 60 min onwards with 30 μM allicin (*p*<0.001).

### Allicin does not induce externalization of Annexin 5 binding membrane phospholipids in *Leishmania*, an early marker of apoptosis

**Fig 9:**
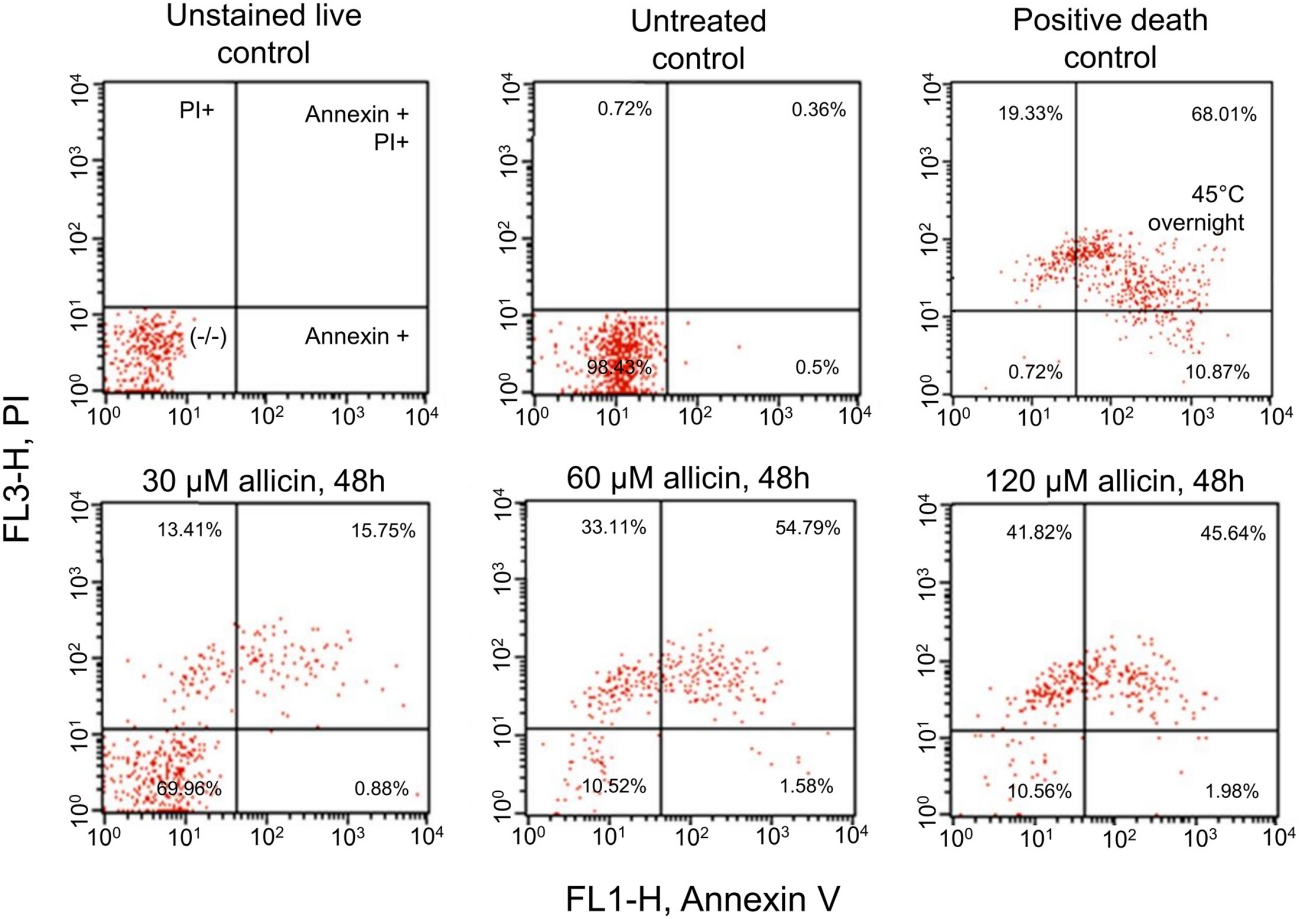
Phospholipids externalization in *Leishmania* promastigotes is not detected with Annexin V and PI (propidium iodide) after treatment of promastigotes with allicin. *L*.*infantum* promastigotes were exposed to allicin (30, 60 and 120 μM) for 3, 12, 24 and 48 h and subsequently stained with Annexin V-FITC + PI and analyzed by flow cytometry. Data correspond to the samples exposed for 48 h. Lower row shows the allicin-treated cultures and the upper row includes unstained cells, untreated control cells and cells exposed overnight at 45°C. Percentages are shown in each quadrant. (-/-): healthy cells; Annexin+: apoptotic cells; Annexin+/PI+: late apoptotic or early necrotic cells; PI+: necrotic cells.

One of the most frequently used early markers of apoptosis is the externalization of phosphatidylserine (PS) by apoptotic cells. It has been shown that *Leishmania* promastigotes lack PS although other phospholipid classes are present and could bind Annexin V upon permeabilization [40]. Fig 9 shows the results obtained, by FACS, after 48 h treatment with 30, 60 and 120 μM allicin. Results obtained in the first three determinations (3, 12 and 24 h) did not yield significant results. Using the EC_50_ concentration of the compound (30 μM) a shift of the cell population from the bottom left quadrant (-/-), characteristic of untreated healthy control cells, towards the upper left (PI+) and upper right quadrants (Annexin +/PI+) was observed. This shift was increased with higher concentrations of allicin and only a residual population remained double negative even with 120 μM. It is noteworthy to indicate that no Annexin single positive (Annexin V+) population was found with any of the concentrations and times of treatment.

### Treatment with allicin of *Leishmania* promastigotes is not followed by DNA fragmentation

**Fig 10:**
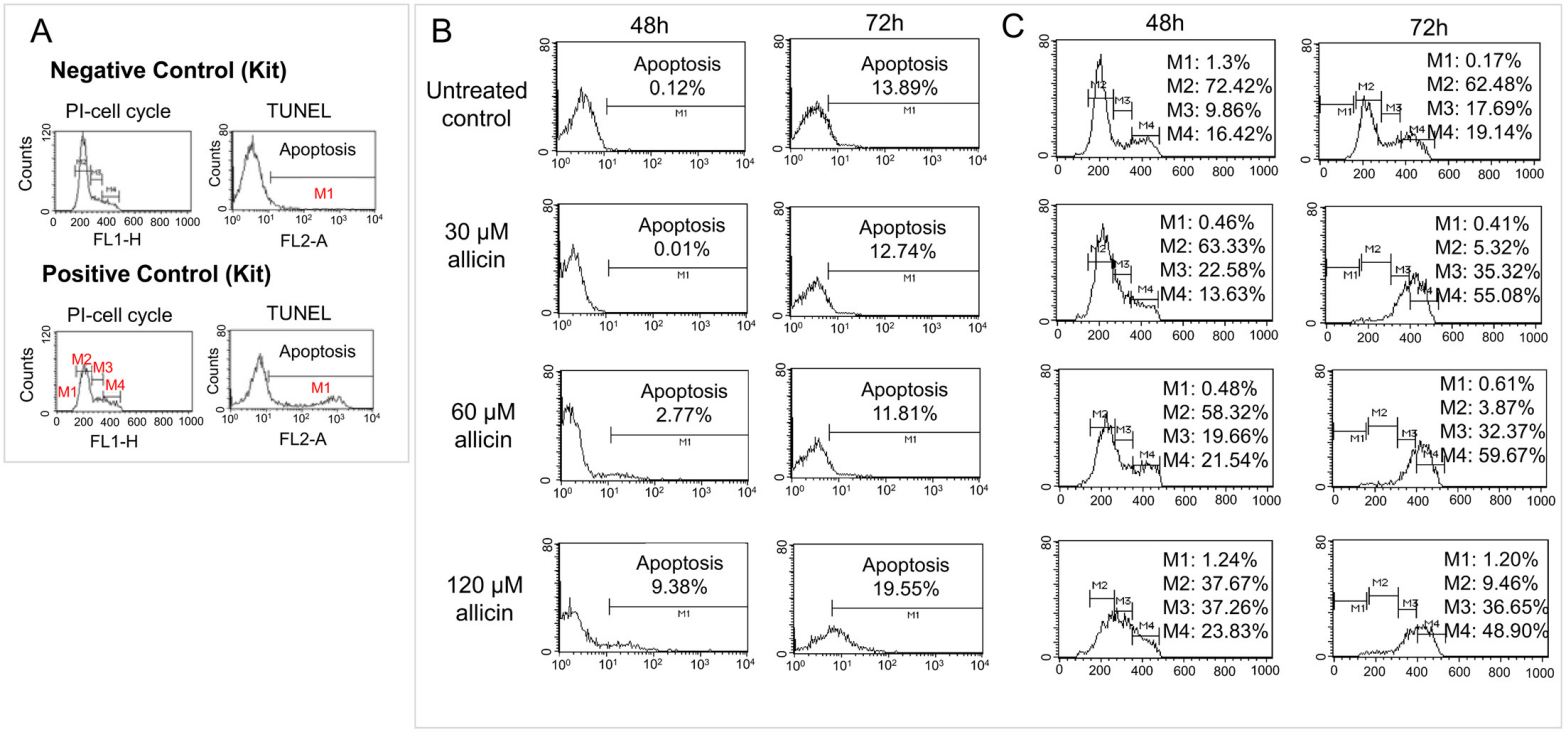
Allicin does not provoke oligonucleosomal DNA fragmentation but it induces cell cycle arrest at G_2_/M phase in *Leishmania*. A, Apoptotic control cells of APO-BrdU TUNEL Assay Kit. B, analysis of DNA fragmentation in *Leishmania*. Promastigotes of *L*.*infantum* treated with allicin (30, 60 and 120 μM) for 48 and 72h and the incorporation of bromodeoxyridine (BrUD) (TUNEL) analyzed by flow cytometry with Alexa Fluor 488 conjugated anti-BrDU antibody. Untreated cell cultures, positive and negative controls were included. C, Cultures of *L*.*infantum* promastigotes treated with allicin (30, 60, 120 μM) (48 and 72h) from the TUNEL assay were incubated with PI/RNAse staining solution and DNA contents and cell cycle progression determined. Untreated promastigotes were included as controls. Cell cycle phases: M1 = Sub-G_1_ (apoptotic cells); M2 = G_0_/G_1_ [diploid chromosome content (2N); growth and preparation for DNA synthesis]; M3 = S (DNA replication) and M4 = G_2_/M [double diploid (4N); G_2_: preparation for mitosis and growth and M: mitosis and cytokinesis].

No intranucleosomal DNA fragmentation was evident in treated cells with allicin using the dUTP nick-end labelling (TUNEL) method and FACS analysis of cultures of *Leishmania* promastigotes treated for 48 and 72 h with allicin (30, 60 and 120 μM) (Fig 10A and 10B). There was a shift in the FL1-H axis but this shift was present in all cultures irrespective of the treatment and the concentration of allicin. A small and possibly non-significant apoptotic population was observed after 48 h in the cultures with the highest allicin concentration (>60 μM). In the 72h sample a significant fraction of the population displayed an apparent phenotypic fragmentation pattern. However cellular distribution was similar in both treated and untreated control cultures.

**Fig 11:**
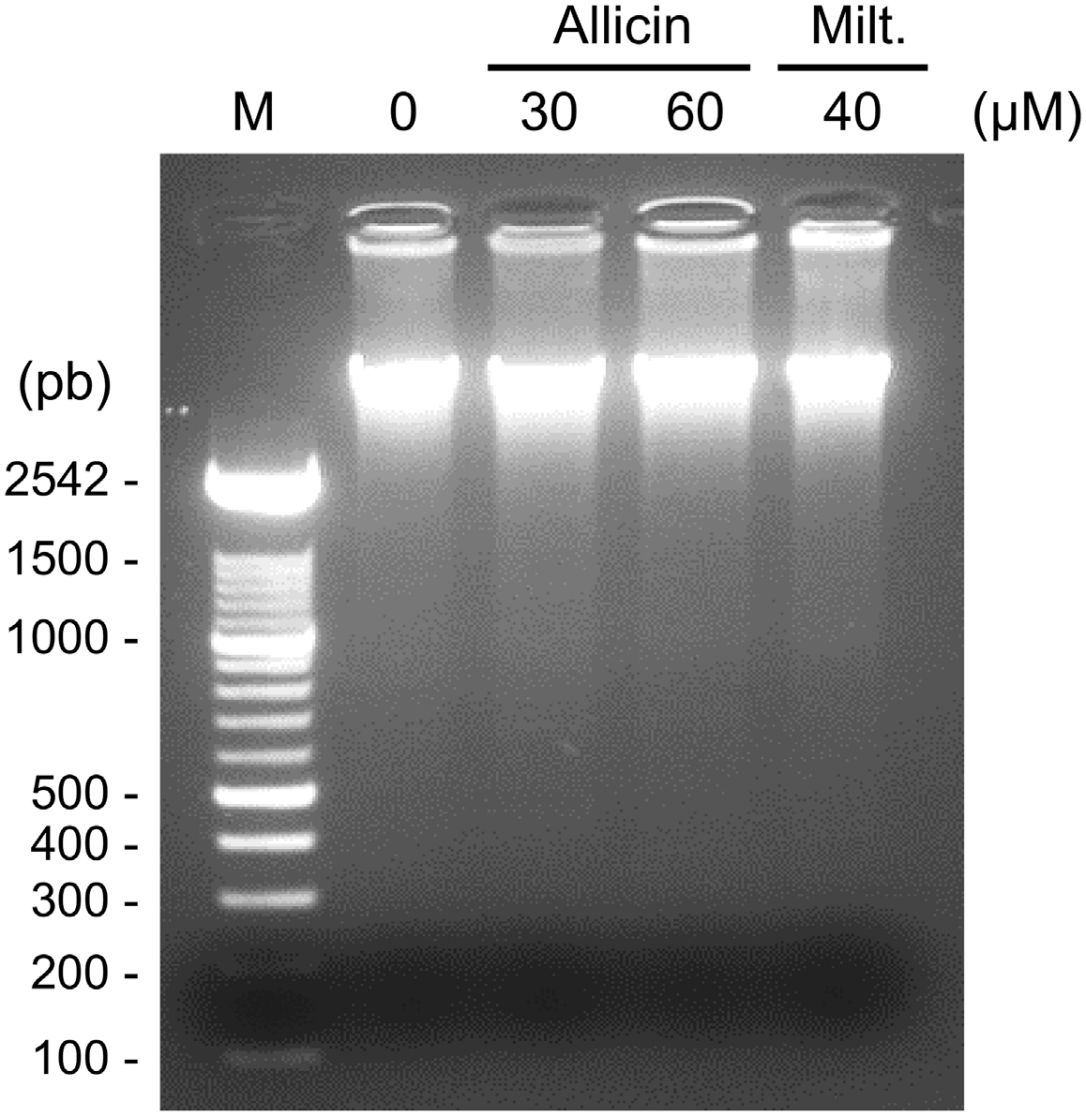
DNA fragmentation analysis by agarose gel electrophoresis of allicin-treated promastigotes of *Leishmania*. DNA profiles from untreated (0), allicin-treated (30 μM and 60 μM) or miltefosine-treated (40 μM) *L*. *infantum* promastigotes after 24 h of incubation at 27°C. M, molecular size marker (bp, base pairs).

Under our conditions neither allicin (30 and 60 μM) nor miltefosine (40 μM) induced any DNA laddering after incubation for 24, 48 or 72 h. Fig 11 shows a representative analysis after 24 h exposition to allicin. *Leishmania* DNA from promastigotes treated with both compounds did not show evidence of apoptotic-like laddering.

### Allicin induces G_2_/M phase cell cycle arrest of *Leishmania* promastigotes

To determine the effect of allicin on the progression of the cell cycle of *L*.*infantum*, promastigotes were treated with different concentrations of the antileishmanial (30, 60 and 120 μM) at different exposure times (48 and 72 h) and analyzed by flow cytometry using PI staining. Allicin induced notable changes in the distribution of cell cycle phases of cultures. These alterations were time and dose dependent as shown at Fig 9C. FACS analysis of untreated control cultures showed 1.3% of cells in Sub-G_1_ phase in 48 h cultures and 0.17% in 72 h cultures. Allicin treatment did not induce the appearance of the Sub-G_1_ peak, characteristic of apoptotic cells, as the highest concentration produced a minimal change in the G_0_/G_1_ phase % (i.e. 48 h: 1.24% and 72 h: 1.20%). Untreated controls presented a large population in the G_0_/G_1_ phase (i.e. 48 h: 72.42% and 72 h: 62.48%) whereas the allicin treated cells showed a dose dependent increase in the G_2_/M phase cell population and a reduction of the cells in G_0_/G_1_ phase. Allicin 120 μM (3 h) induced a marked shift in the G_2_/M gate (i.e. 48 h: 23.83% and 72 h: 48.90%). Results obtained with early (Annexin V binding), late (DNA fragmentation) and cell cycle analysis did not support an apoptotic-like effect of allicin on *Leishmania* but rather cell necrosis.

## Discussion

Our results showed that allicin, at sublethal concentrations (ca. EC_50_), induced an elevation of intracellular Ca^2+^ levels. In most eukaryotic cells a major signaling function of this cation in the cytosolic compartment is played when its levels are elevated. In *Leishmania* Ca^2+^ is maintained at very low levels [41] and the fine tuning of its intracellular levels is critical for cell homeostasis [42]. The high levels of intracellular Ca^2+^ observed in *Leishmania* exposed to allicin probably came from intracellular calcium stores and particularly mitochondrion, since no variations in the plasma membrane permeability were found at least as assessed by SYTOX Green internalization. There is a tight connection between oxidative stress and intracellular Ca^2+^ in all organisms including *Leishmania* [43–45]. Although ROS are present in normal cells playing a significant role as signaling messengers [24] the overproduction is linked to oxidative stress, mitochondrial dysfunction and cell death [46, 47]. Oxidative stress can disrupt the intracellular calcium translocation [48] among calcium stores (e.g. mitochondrion, acidocalcisomes, endoplasmic reticulum). Our results showed that 30 μM allicin induced an increase of mitochondrial and cytosolic ROS levels. To cope with the damaging oxidative stress *Leishmania* relies mainly on trypanothione (= bis glutathionyl spermidine) [49] since these Kinetoplastida are devoid of catalase and classical selenium containing GSH peroxidase and no glutathione reductase is present [50]. However, trypanothione reductase (TryR) activity was only moderately inhibited in the presence of allicin and, expectedly, thiols (cysteine, glutathione and trypanothione) levels increased. These findings, besides confirming the interaction of allicin with thiols [19], suggested that the molecule did not provoke a collapse of the trypanothione reducing system and therefore cell death of allicin-treated *Leishmania* was not primarily related to this mechanism of action.

ROS generation and increase of cytosolic Ca^2+^ were accompanied by the fall of mitochondrial membrane potential (ΔΨm) and reduced ATP production in the fixed time (3 h) experiments carried out. The high levels of superoxide anion (O_2_^-^) inside the mitochondrion and their apparent saturation (> 30 μM allicin) besides the extensive morphological alterations observed in TEM suggested that this organelle could be the primary target of allicin. However, TEM results were obtained with promastigotes exposed to high allicin concentrations for 24 h. Allicin can easily cross cell membranes [19] and surely these events should take place very rapidly after exposition to the molecule. Time course studies carried out did show a rapid and significant elevation of cytosolic Ca^2+^ after 5 min incubation with 60 μM allicin and a 20% increase with 30 μM after 15 min. This elevation was followed by a parallel fall of ATP production, mitochondrial membrane depolarization, and superoxide levels whereas a delayed and non saturated cytosolic ROS production was found up to 3 h. Calcium is a key regulator of mitochondrial function and acts within the organelle to stimulate ATP synthesis [51]. This is critical since an estimated 70% of all energy requirements in *Leishmania* are fulfilled by oxidative phosphorylation in mitochondria [35] and they have a single mitochondrion and poor ability to multiply in anaerobic environments [28, 29]. In contrast to other trypanosomatids TCA cycle in *Leishmania* promastigotes has major anabolic function by anaplerosis and glycosomal and mitochondrial metabolism is tightly coupled [52]. No determination of the glutamate /glutamine ratio in allicin-treated cells has been carried out by us and the involvement of glycosomes in the mechanism of action could also be considered. However, mitochondrial oxidative ATP generation, but not the glycolytic ATP generation, is inhibited by sodium azide [35] and in our time course experiments 30 μM allicin reduced ATP production by 30% and 60% after 15 min and 1 hour incubation, respectively. Thus our results suggest that allicin induced an increase in cytosolic Ca^2+^ levels this leading to high superoxide levels, ΔΨm depolarization with dysfunction of oxidative phosphorylation and subsequent reduction of ATP mitochondrial synthesis. These interconnected events induce a mitochondrial collapse with swelling and loss of organelle integrity and, finally, a cellular energetic catastrophe. Nonetheless, there are open questions and further investigation to validate the proposed mechanism of action is needed. Increase in cytosolic Ca^2+^ due to the influx from the extracellular milieu cannot be excluded and experimentation using calcium chelating agents and antioxidants prior to or in conjunction with the allicin treatment is needed.

Mitochondrial membrane collapse (MMC), and consequently the lack of functionality of mitochondria, is an irreversible cellular dysfunction leading, depending on the relative weight of the bioenergetic catastrophe/protease and endonuclease activation, to cell necrosis or apoptosis respectively [53]. Apparently allicin is able to induce apoptosis in some cancer cell lines (L-929, SGC-7901) [23, 24]. ATP availability, after mitochondrial membrane depolarization, determines the switch from apoptosis (no fall of ATP) to cell necrosis (low ATP) [54–56]. In our case there was a net fall of ATP production. Some apoptotic-like deaths have been described in *Leishmania* [44, 57] although cell deaths mechanisms in parasitic protozoa, in particular apoptosis-like, are a controversial issue [58]. Despite the variations found in programmed cell death our results did not show any conclusive evidence of apoptotic-like death since DNA fragmentation was not observed (DNA laddering, TUNEL assay), no phospholipids (Annexin V/PI staining) were exposed and there was a cell cycle arrest at G_2_/M phase (PI staining). On these grounds, the death type found in *Leishmania* treated with allicin is compatible with cell necrosis.

Additional experiments would precisely determine the relative role of the events and the participation of other organelles but available data point towards the rapid induction of high Ca^2+^ levels and mitochondrial ROS in *Leishmania* by exposure to allicin. The impairment of the redox balance induced the mitochondrial membrane depolarization (ΔΨm) with dysfunction of TCA and reduction of ATP production. These events, possibly through a feed-back process, lead to the collapse of mitochondrion with loss of integrity and finally a cellular energetic catastrophe leading to the necrotic death of *Leishmania* promastigotes with cell arrest at the premitotic phase (G_2_/M). Present results were obtained with promastigotes and further research should confirm that these mechanisms are also involved in the antileishmanial activity of allicin on the actual stage causing leishmaniasis, amastigotes.
